# Equalities analysis of post-pandemic results in general and vocational and technical qualifications

**DOI:** 10.23889/ijpds.v8i2.2309

**Published:** 2023-09-14

**Authors:** Ming Wei Lee, Dovile Rama, Kathryn Johnson

**Affiliations:** 1 Ofqual, Coventry, United Kingdom

## Objectives

We exploited linked administrative data to study attainment gaps in GCSEs, A level, and vocational and technical qualifications. Our aim was to compare how grades for groups of students with different protected characteristics and socio-economic status have changed in 2022 compared to previous years.

## Methods

We used a multilevel regression modelling to evaluate the impact of each demographic and socio-economic characteristic on students’ results, once other factors are controlled for. The purpose was to examine how the relationship between students’ attainment and their characteristics has changed between 2018 and 2022. The characteristics analysed were:

- ethnicity
- gender
- special educational needs and disabilities status
- free school meal eligibility
- Income Deprivation Affecting Children Index score
- prior attainment
- first language
- region
- centre type

We collected results and prior attainment data from awarding organisations and linked it with data from the Individual Learner Record and the National Pupil Database.

## Results

Of the many different comparisons between groups of students, the majority showed no notable change in attainment gaps in 2022, compared with both pandemic and pre-pandemic years. Some degree of minor fluctuation is always to be expected.

Attainment gaps and changes over time differ across qualifications. At A level, the analysis highlighted some key changes in results in relation to gender (the difference between boys and girls reversed during the pandemic), ethnicity and school and/or college type.

At GCSE, the analysis showed notable changes for ethnicity, with Gypsy and Roma students narrowing the gap compared to White British students, school and/or college type and socio-economic background.

For vocational and technical qualifications, we found fewer notable changes than for GCSEs and A levels.

## Conclusion

The use of linked administrative data helped to gain better insights in attainment gap changes over time. Many gaps remained the same, some gaps widened, and others narrowed. The findings make it difficult to draw firm conclusions about the cause of these differences, many of which existed before the pandemic.

